# A simplified sagittal split osteotomy of the mandibular ramus: A single-cut approach for orthognathic surgery

**DOI:** 10.4317/medoral.27649

**Published:** 2025-10-17

**Authors:** Samuel Macedo Costa, Marcelo Santos Bahia, Marcella Yumi Kadooka, Priscila Faleiros Bertelli Trivellato, Cassio Edvard Sverzut, Alexandre Elias Trivellato

**Affiliations:** 1 Department of Oral and Maxillofacial Surgery; School of Dentistry of Ribeirão Preto of the University of São Paulo, São Paulo, Brazil

## Abstract

**Background:**

This study aims to evaluate the feasibility, and reliability of a novel simplified sagittal osteotomy (SSO) technique for mandibular repositioning in orthognathic surgery. The research question focuses on whether this approach can reduce technical complexity and complication rates, especially in a residency training environment.

**Material and Methods:**

A prospective observational study was conducted involving 118 patients (62 females, 56 males) diagnosed with Angle Class II or III malocclusions and indicated for orthognathic surgery. All patients underwent a novel mandibular osteotomy as part of a Double-jaw surgery. Exclusion criteria included patients under 18 years of age, third molar presence, mandibular pathologies, and incomplete follow-up. The primary outcomes included feasibility, complication rates, and the need for conversion to the conventional BSSO. Statistical analysis was performed using the Chi-square test.

**Results:**

A total of 236 osteotomies were performed. The technique was considered feasible in 98.7% of cases, with no conversions to traditional BSSO. No bad splits occurred. IAN injury was observed in 0.8% of cases, and long-term paresthesia (&gt;1 year) occurred in 1.2%. No cases of hemorrhage or dental injury were reported. No significant associations were found between complications and patient gender or deformity type (p&gt;0.05).

**Conclusions:**

The SSO technique demonstrated high feasibility and a low complication profile, supporting its use as a reliable alternative to BSSO.

## Introduction

The Bilateral Sagittal Split Osteotomy (BSSO) is widely recognized as the gold standard for orthognathic surgery of the lower jaw and has undergone numerous refinements over the years (1 - 4). Traditionally, the BSSO involves a basal osteotomy in combination with sagittal segment and ramus osteotomies. First described in 1957 by Trauner and Obwegeser, the technique has since been modified to enhance the stability of this elective procedure (1).

The BSSO typically comprises three osteotomies: The medial ramus, the sagittal cut, and the basal osteotomy. Each of these steps presents potential challenges, particularly for less experienced surgeons and residents (4). The medial ramus osteotomy should be performed above the lingula, following direct visualization made possible by an anterior and medial subperiosteal dissection of the ramus. This dissection is technically demanding and may result in hemorrhage from the medial pterygoid muscle or, more critically, damage to the inferior alveolar nerve (IAN) prior to its entrance at the mandibular foramen (4).

The sagittal cut is a crucial step for achieving an appropriate mandibular split and should be positioned centrally along the buccolingual crest. It must be directed through the cortical bone, parallel to the molars, with great care to avoid injuring the IAN. In the conventional technique, this cut can be performed using burrs, a saw, or piezoelectric instruments, and must be deep enough to allow the insertion of an osteotome or chisel. Mistakes at this stage can compromise the entire procedure; for example, a thin buccal cortical plate may increase the likelihood of an unfavorable split of the proximal segment during osteotome manipulation (1 , 5).

The final step-the buccal osteotomy including the basal segment-is particularly critical. The osteotomy should begin at the mandibular base, ensuring that both sides are cut to prevent a J-shaped split ("J-split") (6), and then continue along the vertical buccal segment to connect with the sagittal osteotomy. It is essential that the basal segment is fully separated and that the vertical cut is precisely placed through the cortex, as the instrument lies perpendicular to the IAN.

Although complications with BSSO are uncommon, the procedure remains challenging, especially for less experienced professionals and in educational environments such as residency training. Each step requires precision and anatomical understanding to avoid unfavorable outcomes.

In this study, we propose and validate a novel concept that simplifies the osteotomy process by eliminating angular cuts and employing a single sagittal cut. This design facilitates mandibular movement, reduces technical complexity, minimizes the risk of a "bad split," and better preserves the integrity of the inferior alveolar nerve.

## Material and Methods

Study Design and Sample

This prospective observational study evaluated the feasibility, reliability, and safety of a single sagittal mandibular osteotomy, conducted within an Oral and Maxillofacial Surgery Residency Program, with a follow-up period of 24 months. The study was conducted in accordance with the principles of the Declaration of Helsinki and adhered to the STrengthening the Reporting of OBservational studies in Epidemiology (STROBE) guidelines (6).

The study included all patients presenting with dentofacial deformities who had both the desire and clinical indication for orthognathic surgery, including individuals with Angle Class II and Class III malocclusions, starting from 2017.

Exclusion criteria included patients under 18 years of age, single-jaw maxillary surgery, loss to follow-up before 24 months, revision surgeries, history of mandibular infection, presence of mandibular third molars, and presence of cystic or tumoral lesions.

The final sample included 118 patients (62 females and 56 males). All underwent comprehensive orthodontic preparation, standardized imaging protocols, virtual surgical planning, and a single sagittal mandibular osteotomy to achieve the planned mandibular repositioning. Le Fort I osteotomy was performed as routinely indicated.

All the patients provided the informed consent to participate in this study, in accordance with the helsinki protocol and the irb standards.

-Orthodontic Preparation and Pre-op Setup

Orthodontic treatment was planned collaboratively with the orthodontic team through scheduled interdisciplinary meetings. All patients received standardized treatment using conventional orthodontic appliances. Surgical readiness was determined based on a comprehensive evaluation of occlusal models, clinical measurements, and photographic analysis. Final approval for surgery was granted only after consensus was reached between both the orthodontic and surgical teams.

Single-Sagittal Osteotomy Technique

The procedure begins with a standard incision and exposure technique, extending from the first molar to the lateral aspect of the mandibular ramus, with an average length of 15 to 20mm. Soft tissue dissection is kept to a minimum, limited to exposure of the osteotomy line and the anterior base of the planned cut. There is no need for extensive dissection of the mandibular ramus, either buccally or lingually. A ramus retractor is placed, and the osteotomy is initiated at the medial aspect of the mandibular ramus, at the junction of the buccal and lingual cortices, using a reciprocating saw.

**Figure 1:**
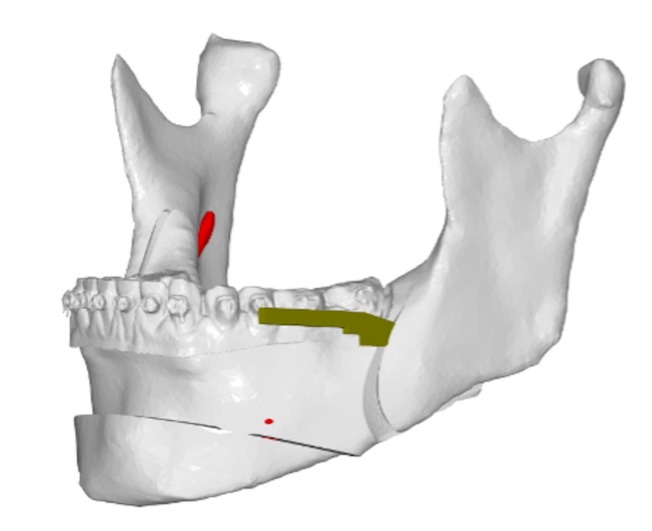
Single-sagittal split osteotomy representation, with the representation of the mandibular sagittal cut and deepening of the reciprocating saw in the software NEMOFAB (Nemotec, Spain).

The cut proceeds linearly beneath and parallel to the oblique line, with the saw oriented parallel to the buccal cortex and extending toward the distal surface of the second molar, in a more buccally oriented trajectory than the conventional BSSO. Importantly, the inclination of the saw remains constant throughout the single sagittal cut, thereby minimizing the risk of injury to the inferior alveolar nerve (IAN). The cut continues anteriorly and buccally; once the desired extension is reached, it is directed toward the buccal cortex to complete the osteotomy. If necessary, the saw may be slightly everted to reach the base of the anterior cut area without increasing the dissection area (Figure 1).

1

**Figure 2:**
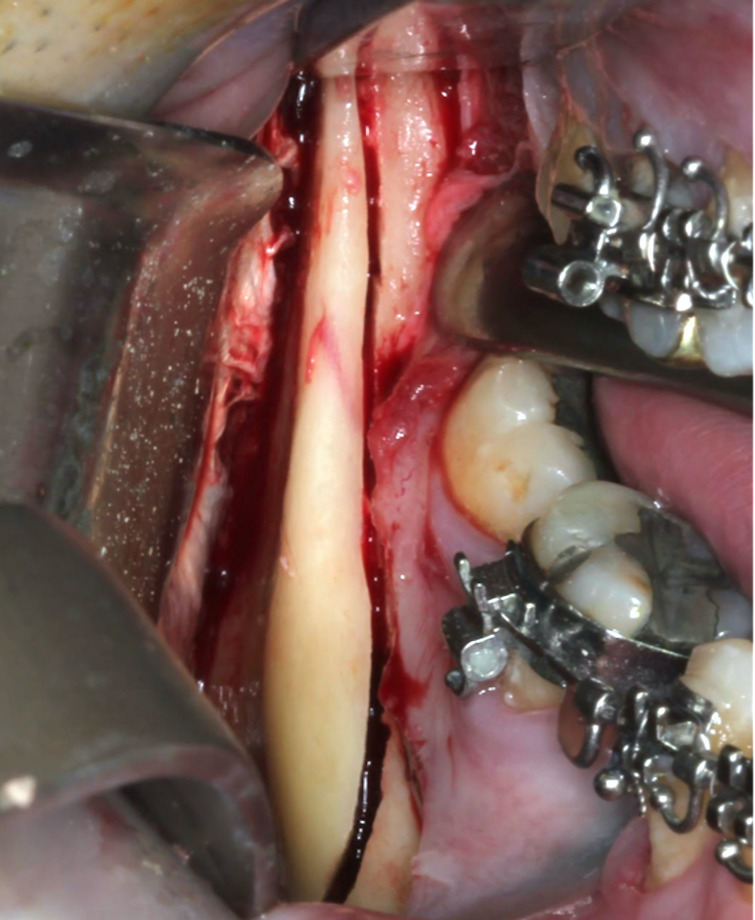
Clinical photography of the single-sagittal split osteotomy and its complete section.

Segment separation is achieved using a separator, with gradual force applied to the mandibular base-first at the anterior region of the osteotomy and then at the distal aspect of the second molar. This technique eliminates the need for chisels, thereby reducing procedural trauma. It also significantly lowers the risk of IAN injury (Figure 2). Following segment mobilization, the IAN is protected when necessary, and mandibular repositioning and osteosynthesis are carried out according to the preoperative surgical plan.

2

Depending on the planned mandibular movement, the osteotomy length may vary, while still allowing for a sliding motion suitable for both advancements and setbacks. If necessary, the technique can be easily converted to the conventional BSSO, by applying the basal osteotomy and/or the ramus osteotomy. The need for conversion was recorded in this study.

Evaluation of Feasibility and Reliability

To assess the feasibility of the SSO, a qualitative and quantitative survey-based evaluation was conducted among in-training oral and maxillofacial surgeons and residents. A third-party professional, independent from the surgical team, was responsible for administering the evaluation and collecting the data to ensure unbiased responses. All surgeons and residents who directly participated in the procedure using the novel osteotomy technique were invited to complete the evaluation. The participants were asked to answer if the SSO was easy to execute, if the anatomical landmarks were easy to identify, the perceived safety of the technique, and general satisfaction. If the professional felt the SSO was feasible and reliable, the answer was recorded.

Statistical Analysis

The Chi-square test was selected to analyze associations between categorical variables, such as gender, dentofacial deformity classification, and the presence of complications. This non-parametric test is appropriate for evaluating relationships between independent variables in contingency tables, particularly with frequency data. Demographic variables were also collected and analyzed to explore potential correlations. All statistical analyses were performed using IBM SPSS Statistics software (version 24), ensuring reliable and standardized processing of the dataset.

## Results

A total of 118 patients (236 osteotomies) underwent the single sagittal osteotomy (SSO) procedure. The sample was composed of 62 females (52.5%) and 56 males (47.5%). The majority of patients presented with Class II dentofacial deformity (82 cases, 69.4%), while Class III deformities were observed in 36 patients (30.6%).

Regarding feasibility, the SSO was considered feasible in 233 out of 236 osteotomies (98.7%). Only three osteotomies (1.3%) were deemed not feasible by the evaluating professionals. Additionally, none of the cases required intraoperative conversion to the conventional bilateral sagittal split osteotomy (BSSO), suggesting a high procedural reliability.

Analysis of complications revealed a low incidence across all categories. No bad split was reported, while inferior alveolar nerve (IAN) damage occurred in two osteotomies (0.8%). Extended IAN paresthesia, defined as persisting for over one year, was observed in six cases (1.2%). Notably, no instances of hemorrhage or dental injury were reported.

When stratified by gender, complications were slightly more frequent among male patients, although statistical analysis using the Chi-square test revealed no significant correlation between gender and the occurrence of complications (p&gt;0.05). Similarly, when assessing the relationship between deformity type and complications, no statistically significant association was identified (p&gt;0.05), suggesting that neither deformity class nor patient gender substantially influenced complication rates in this sample. All data is displayed in Table 1.

Table 1

## Discussion

This study introduces and evaluates a novel single-sagittal osteotomy (SSO) technique for mandibular repositioning in orthognathic surgery, offering a simplified alternative to the conventional bilateral sagittal split osteotomy (BSSO)(4). The traditional BSSO, while effective and widely adopted, carries inherent technical challenges-particularly related to the anatomical complexity of the mandibular ramus and the risk of complications such as bad splits and inferior alveolar nerve (IAN) injury (4 , 5 , 6). These risks are notably higher in residency training contexts where less experienced surgeons perform the procedures.

The SSO technique proposed here is a linear cut that minimizes soft tissue dissection and eliminates the need for chisels and multiple angular osteotomies. By maintaining a consistent saw inclination and avoiding the basal and medial ramus cuts typical of BSSO, this technique reportedly decreases the likelihood of IAN injury and simplifies the overall procedure, which could be suitable for the training context.

Results from 118 patients (236 osteotomies) support the feasibility (98.7%) and reliability (0% need for conversion to BSSO) of the SSO. Additionally, complication rates were low: No cases of bad split, only 0.8% IAN injury, and 1.2% of long-term paresthesia. These findings are promising, especially in a teaching setting, suggesting that the SSO could represent a safer and more accessible option for orthognathic procedures in selected patients.

**Figure 3:**
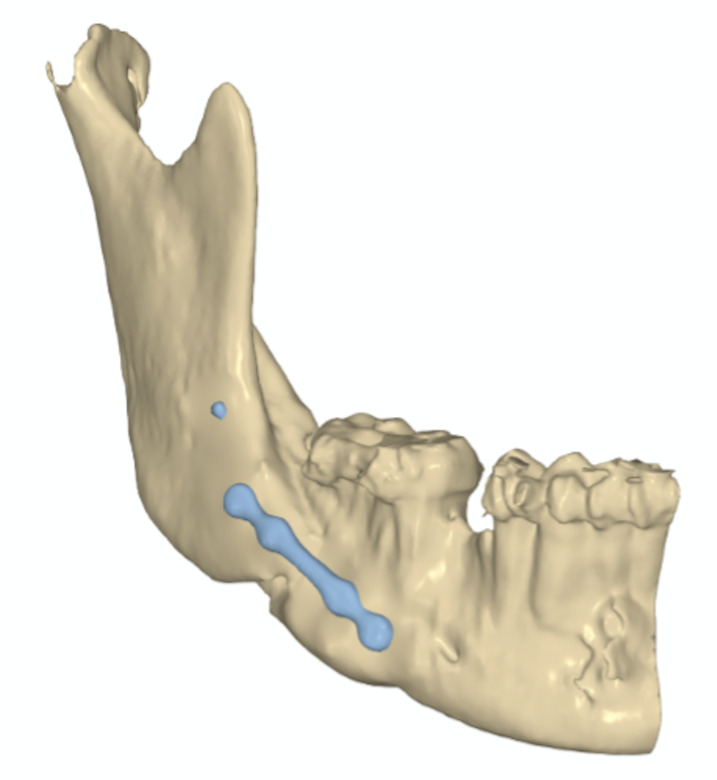
Virtual representation of the fixation in the postoperative of a 10mm advancement, with reduced notch, in Software NEMOFAB (Nemotec, Spain).

This osteotomy design avoids the formation of a non-aesthetic mandibular notch. Bone contouring between separated segments is rarely required. This osteotomy allows for a wide range of fixation options. Within our team, a hybrid fixation technique is commonly utilized (Figure 3); however, two-plate or screw-only fixation methods are also feasible, primarily due to the extensive contact area between the segments.

3

Additionally, due to its straightforward nature, this osteotomy is easily reproducible by residents and less experienced clinicians, thereby reducing the number of procedural steps and the need for specialized instruments such as osteotomes, mallets, and special separators.

However, while the data are encouraging, the study is limited by its observational design and lack of a control group. A comparative analysis between SSO and BSSO in terms of operative time, postoperative function, neurosensory outcomes, and long-term skeletal stability would strengthen the clinical validity of the proposed method. Additionally, the inclusion criteria omitted more complex cases (e.g., revision surgeries, third molar presence, mandibular pathology), which may affect generalizability.

## Conclusions

The single-sagittal osteotomy technique represents a viable, simplified alternative to the conventional BSSO for orthognathic mandibular surgery. By reducing the number of osteotomy steps, the SSO enhances surgical efficiency and decreases the risk of complications-particularly inferior alveolar nerve injury and bad splits. The high feasibility and low complication rates observed in this study underscore the technique's safety and reliability, especially within a residency training environment. While further controlled studies are needed to compare long-term outcomes with conventional methods, the SSO emerges as a promising option for improving both the learning curve and surgical outcomes in orthognathic procedures.

## Figures and Tables

**Table 1:** Table Descriptive and statistical analysis of the patients.

Variable	Total	Statistics Analysis+
Total Patients	118	
Total Osteotomies	236	
Gender		
Male	56 (47,5%)	-
Female	62 (52,5%)	-
Deformity		
Class II	82 (69,4%)	0,333
Class III	36 (30,6%)	
Considered Feasible		
Yes	233(98,7%)	-
No	3 (1,3%)	-
Need for Conversion for Traditional BSSO		
Yes		-
No	236 (100%)	-
Complications		
Bad-Split	0	-
IAN Damage	2 (0,008%)	-
>1yr IAN Paresthesia	6 (0,012%)	0,907
Hemorrhage	0	-
Tooth Injury	0	-

*Chi-Square Test. P<0,05 considered statistical significant.

## Data Availability

Declared none.
